# Novel intralesional nemolizumab for chronic scalp prurigo nodularis

**DOI:** 10.1016/j.jdcr.2026.06.005

**Published:** 2026-06-26

**Authors:** Emily Gaven, Sarah Siddiqui, Molly Stout

**Affiliations:** SkinCare Physicians, Chestnut Hill, Massachusetts

**Keywords:** itch, nemolizumab, prurigo nodularis, scalp pruritus

## Introduction

Prurigo nodularis (PN) is a chronic inflammatory dermatologic disorder characterized by intensely pruritic nodules, commonly affecting the trunk, extremities, and scalp. Lesions are typically firm, dome-shaped, and vary in color from flesh-colored to erythematous. The disease predominantly affects adults between 40 and 69 years of age and has been associated with significant impairment in quality of life due to persistent pruritus and sleep disturbance.^1^ Lesions consistent with PN often arise from repetitive scratching of initially normal-appearing skin, resulting in a self-perpetuating itch-scratch cycle and progressive nodule formation.^2^

Traditional management includes topical and intralesional corticosteroids, neuromodulators (eg, gabapentin), and systemic immunosuppressants; however, many patients experience incomplete or transient responses.^1^^,^^3^ Targeted biologic therapies have shifted treatment toward specific immune pathways. Dupilumab, an IL-4 receptor α antagonist, has demonstrated efficacy in reducing pruritus.^4^ More recently, nemolizumab has expanded treatment options by directly addressing the interleukin 31 (IL-31)–mediated neuroimmune pathway driving chronic itch.^5^^,^^6^

Nemolizumab (Nemluvio) is a biologic agent approved for the treatment of atopic dermatitis and PN. It selectively targets the IL-31 receptor A, a key mediator of chronic pruritus and neuroinflammatory signaling in inflammatory skin diseases.^6^ IL-31, a T-helper 2–derived cytokine, plays a central role in the pathogenesis of PN by activating neuronal itch signaling and promoting the itch-scratch cycle characteristic of PN, ultimately contributing to nodule formation.^7^^,^^8^ By blocking IL-31 receptor signaling, nemolizumab disrupts the neuroimmune feedback loop, reducing pruritus and inflammation, leading to clinical improvement.^5^^,^^6^

Nemolizumab is approved for systemic subcutaneous administration and has demonstrated significant reductions in itch scores and lesion counts in clinical trials.^5^ To our knowledge, intralesional administration has not previously been reported. We present a novel case of intralesional nemolizumab use for localized scalp PN.

## Case presentation

A 56-year-old man presented with a 10-year history of chronic pruritus consistent with scalp-localized PN. Prior treatments included oral antibiotics (cephalexin 500 mg daily) and approximately 20 intralesional triamcinolone acetonide (Kenalog) injections, with minimal benefit. He presented in December 2025 with severe pruritus significantly impairing quality of life (Fig 1).

**Fig 1:**
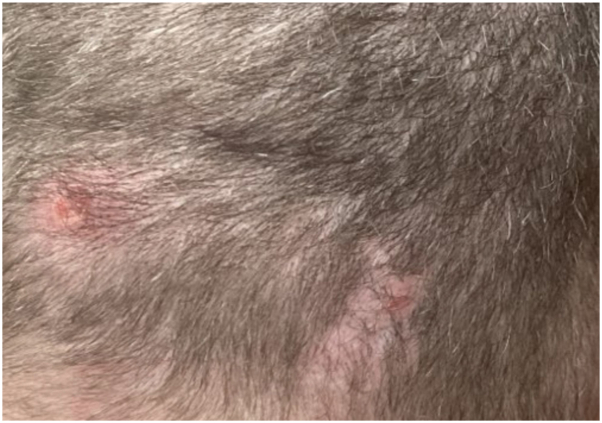
Clinical presentation of localized prurigo nodularis of the scalp before intralesional nemolizumab injection, demonstrating multiple erythematous hyperkeratotic nodules with central erosion and surrounding inflammation.

Given the refractory nature of disease and the need to disrupt the itch-scratch cycle, nemolizumab was selected due to its rapid onset of action. As the medication is only available as a dual-chamber autoinjector intended for subcutaneous administration, modification was required. Under sterile conditions, including the use of gloves and sterile instruments, the autoinjector discharged into a sterile urine cup and nemolizumab was immediately drawn into a 1-mL Luer lock syringe.

Based on labeled concentration, approximately 25 mg (0.5 mL) was administered intralesionally, divided between 2 occipital nodules (0.25 mL per lesion). Exact dose and concentration could not be independently verified following extraction, and minor loss during handling is possible. The solution was handled gently and administered immediately to preserve protein stability.

At 1-month follow-up, the patient reported marked improvement in symptoms, with pruritus decreasing from 8/10 to 1/10 on a numerical rating scale and sleep disturbance improving from 7/10 to 3/10. Clinical examination demonstrated a reduction in nodularity and excoriation (Fig 2). Two occipital scalp nodules were initially identified, measuring 12 mm (left) and 7 mm (right), both of which showed interval regression at follow-up. This response represented the most significant therapeutic benefit achieved compared with prior interventions. A prurigo nodule on the thigh, which was not treated, showed no improvement.

**Fig 2:**
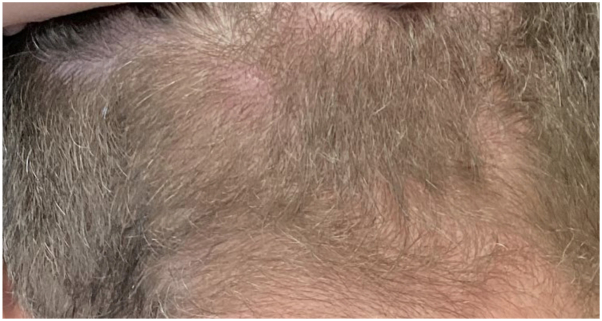
Clinical appearance of the scalp 1 month after intralesional nemolizumab injection, demonstrating substantial regression of nodules and decreased inflammation.

The patient was subsequently transitioned to subcutaneous nemolizumab 30 mg monthly. Following successful disruption of the itch-scratch cycle with intralesional nemolizumab, transition to a standard subcutaneous dosing regimen was recommended to optimize treatment efficacy and improve ease of administration. At 2-month follow-up, he reported complete resolution of pruritus (numerical rating scale score, 0/10) with continued improvement in inflammation.

## Discussion

PN remains difficult to manage, particularly in localized, treatment-refractory cases.^2^^,^^5^ In this case, intralesional nemolizumab produced rapid and substantial symptom relief after failure of conventional therapies. The patient reported improvement within 72 hours, with pruritus decreasing from 8/10 to 1/10 at 1-month follow-up, suggesting that localized IL-31 receptor blockade may exert a direct and immediate effect on neuronal itch signaling at the site of pathology.^5^^,^^6^

Despite these findings, several limitations must be acknowledged. This is a single patient with short-term follow-up, limiting generalizability. Additionally, transition to systemic nemolizumab shortly after treatment confounds interpretation of long-term durability, as sustained improvements may reflect combined effects.

The rapid onset of improvement observed in this case is notable. IL-31 acts directly on peripheral sensory neurons expressing IL-31 receptor A, promoting neuronal excitability and transmission of pruritus signals.^7^ Localized inhibition of the pathway may attenuate neural sensitization more efficiently at high-density lesional sites, such as the scalp. Additionally, reducing pruritus early may interrupt the self-perpetuating itch-scratch cycle, preventing further mechanical trauma and inflammatory amplification.^1^^,^^7^

Intralesional delivery offers several theoretical advantages, including targeted drug exposure, reduced systemic burden, and potential utility in focal disease.^4^, ^5^, ^6^ By administering therapy at the site of active lesions, it may enhance local efficacy while minimizing systemic adverse effects and overall medication burden. In patients with limited disease, this approach may serve as a targeted alternative to systemic therapy. In this case, intralesional treatment effectively disrupted the itch-scratch cycle, allowing transition to standard monthly subcutaneous maintenance dosing.

However, practical limitations exist. Nemolizumab is currently available only as an autoinjector designed for subcutaneous use.^6^ Intralesional delivery requires device modification and manual extraction, introducing technical challenges, potential dosing variability, and risks related to sterility and biologic stability. Development of alternative delivery systems, such as prefilled syringes for intralesional use, may improve precision, reproducibility, and feasibility. Future studies are needed to evaluate safety and optimal dosing in larger patient cohorts.

In conclusion, this case demonstrates the potential utility of intralesional nemolizumab as a novel and promising therapeutic approach for localized PN and suggests that localized biologic delivery may represent an important future direction in the management of chronic pruritic disorders.

## Conflicts of interest

None disclosed.
